# Exploring the purpose and stages of patient and public involvement and engagement (PPIE) in audiology research: a case study approach

**DOI:** 10.1186/s40900-025-00672-9

**Published:** 2025-01-17

**Authors:** Helen Pryce, Nisha Dhanda, Jean Straus

**Affiliations:** 1 College of Health and Life Sciences, Aston University, Birmingham, UK; 2 College of Health and Life Sciences, University of Birmingham, Birmingham, UK; 3 Patient Researcher and PPI Lead, College of Health and Life Sciences, Aston University, Birmingham, UK

**Keywords:** Audiology, Hearing, Patient and family engagement, Health services research, Patient participation, Patient involvement

## Abstract

**Background:**

Patient and Public Involvement and Engagement (PPIE) has become an integral component of contemporary audiology research. It aims to capture diverse views and experiences, essential for evaluating the long-term impact of technological advancements and care models on individuals. Traditional inclusion methods, such as focus groups, may exclude individuals with additional needs or communication difficulties, necessitating the development of more inclusive approaches.

**Methods:**

This study explores PPIE’s purpose and stages in two recent audiology studies, adhering to the Guidance for Reporting Involvement of Patients and the Public (GRIPP2) criteria. The authors describe their attempts to optimise participation by PPIE volunteers in two studies. The methods sought to engage volunteer PPIE contributors who might otherwise have not known about or contributed views of research methods. Specifically, our novel methods for engagement meant reaching out deliberately to marginalised communities and using individual, relationship building approaches, rather than relying on previously described focus group style meetings. Flexible engagement methods were developed to include marginalised communities with lower levels of research engagement. In the first study, the Hearing Loss and Patient Reported Experience Study (HeLP) developed the first Patient-Reported Experience Measure (PREM) in audiology. The study involved creating a conceptual framework, developing a prototype questionnaire, and validating it through test-retest procedures. The second study conducted an ethnographic analysis of adults with dementia and hearing loss in care homes, employing environmental audits, ethnographic observations, and semi-structured interviews.

**Results:**

The HeLP study successfully developed a culturally meaningful and accessible PREM, capturing the experiences of individuals with hearing loss, including those from South Asian communities. PPIE contributors highlighted the need for non-judgmental, empathetic approaches and culturally sensitive language. The PPIE component of the ethnographic study emphasises the importance of creating appropriate communication environments and implementing tailored inclusion strategies to address barriers faced by PPIE contributors in their participation.

**Conclusions:**

The findings stress the necessity of inclusive PPIE methods in audiology research. Building meaningful relationships with PPIE contributors ensures a wide range of perspectives, informing research design and conduct. The study highlights the challenges of traditional focus groups and the benefits of flexible, personalised engagement methods. By prioritising relationship building and nuanced conversations, researchers can develop interventions that better align with users’ needs, preferences, and lifestyles. This approach contributes to the growing body of literature advocating for more inclusive and representative PPIE practices in health research, ultimately enhancing the relevance and impact of audiology studies.

## Abstract (summary)

This article examines two examples of how patients and the public can be involved in audiology research, to highlight how to optimise such participation and engagement. Patient and Public Involvement and Engagement (PPIE) is essential for capturing diverse perspectives and improving research quality. However, traditional methods for including people, like focus groups, may exclude people with communication difficulties or additional needs. This paper reports on our attempts to develop flexible methods to engage marginalised communities, emphasising the importance of building meaningful relationships with PPIE contributors. Two case studies illustrate PPIE’s role in audiology research. The Hearing Loss and Patient Reported Experience Study (HeLP) developed the first Patient-Reported Experience Measure (PREM) in audiology. It aimed to capture patient experiences with hearing loss and clinical care. PPIE contributors helped shape the questionnaire, ensuring it was relevant and culturally sensitive. An Ethnographic Study of Adults with Dementia and Hearing Loss in Care Homes explored factors contributing to social isolation. The researchers used data from audits, observations, and interviews with residents and staff. PPIE activities included understanding residents’ and staff’s experiences and developing effective communication methods. The study highlights the need for inclusive engagement methods to gather a wide range of views. It emphasises the importance of relationship building and nuanced conversations to inform research design and conduct, ultimately leading to more user-centred and effective audiology research.

## Background

Patient and Public Involvement and Engagement (PPIE) has become a critical component of contemporary research [1]. It is often involved in achieving important outcomes. Outcomes in Involvement mean that patients and members of the public contribute ideas to help guide how research happens; outcomes in Engagement mean that awareness of the research that is going on in a particular field of health is extended. Such outcomes are often viewed as a form of consensus in making decisions. For researchers to agree on a prioritisation of health questions and methods of research for a given topic, it is useful to achieve PPIE consensus.

There are external factors in the UK driving researchers to involve people with health conditions in the design and conduct of research when investigating a health condition [2]. UK standards for public involvement stipulate the importance of reflection and learning to inform research [2]. These standards emphasise the importance of inclusion with a requirement that researchers consider just how inclusive their PPIE activities are, whether barriers to participation have been addressed and whether there is flexibility in the ways people can participate [2].

. Unless we are deliberately seeking a heterogeneous range of views, we run the risk of over-emphasising some views over others. Specifically, we run the risk of over-emphasising the perspectives of people who can afford to volunteer their time, travel to research sites, learn about and access opportunities. This limits the perspectives gained to more affluent, well-educated groups who have reason to value research. It also biases against individuals with communication difficulties, who may find it harder to access opportunities and participate in consensus-building discussions.

Individuals who are affected by a wide range of hearing difficulties will undergo diagnostic and rehabilitative procedures. The hope would be to design interventions that account for the many values and preferences of those they are designed to support and that are well-aligned with the variety of users’ needs, preferences and lifestyles. Therefore, any intervention developed for persons with hearing loss should consider the heterogeneous populations that will potentially uptake interventions.

The rapidly increasing technological development in audiology requires Patient and Public Involvement and Engagement (PPIE) to evaluate the long-term impact of technological use and delegation of care on individuals at every stage of the audiological research process, from basic science to clinical application [3–6].

But before we can include people in the design and conduct of research, it is important to consider the way hearing loss affects people in varying ways. Hearing loss has well-known negative effects on individuals, including a sense of stigma, and this sometimes influences the uptake and use of devices and services in audiology [3–6].

The heterogeneity of the audiology patient population means it is important not only to understand the consensus views of audiological research and practice, but also to factor individual variance into research methods. For example, older people in residential care may have different views to younger adults, teenagers, and children.

We already know that within audiological research, colleagues have provided details of how impactful PPIE has been on their decision making [7, 8]. And yet the dominant strategy for obtaining feedback from PPIE individuals has been the ‘focus group’ style method of working towards a consensus of view on research decisions. Achieving consensus has the advantage of providing access to a range of individuals in an expedient manner and encourages a range of different perspectives in audiological PPIE. Frequently, however, PPIE is sessional with a specific agenda devised by researchers for each meeting or ‘session’ [1]. For people with hearing loss, such group sessions present additional communication challenges, and there is a risk of excluding individuals, not least because it is easy to get lost in conversation. Individuals with additional needs, such as cognitive decline or learning disabilities, require tailored engagement methods. Likewise, cultural sensitivities mean that groups may have to be carefully selected by same-sex PPIE researchers.

The methods for engagement in audiological research need careful consideration to ensure that a wide range of public views are included, including those from marginalised communities with lower levels of engagement in research. In our research, we have therefore tried to develop methods that would be flexible in engaging a wider range of community groups and individuals. These involvement and engagement activities should provide an opportunity for meaningful learning rather than a simple confirmation of researcher-planned processes.

To ensure that these types of more nuanced conversations can take place, it is important to develop meaningful relationships with PPIE respondents. Such relationships have been described in the UK standards as ‘working together’, referencing the need for researchers and PPIE contributors to work together to agree goals, activities, and roles. This can include co design in some studies. To do so, relationships need to be developed fully with contributors rather than just consulting contributors with an expectation of agreement [2].

There is a dearth of reported PPIE involvement in the development of patient-reported outcome measures in audiology. There has been some recent description of using PPIE groups to inform the development of research questions and processes by Boddy et al. (2020) [7], and Studts (2022) [9] described the importance of implementing changes in audiology to engage in a wide range of public stakeholders. Dawes et al. (2022) [8] described the use of PPIE across five audiological research projects, including the targeted involvement of people with autism from South Asian community groups and teenagers. These PPIE descriptions include innovative practices, such as employing researchers who have autism to lead the development of materials.

In this article our aim is to contribute to the growing body of literature by describing the involvement of PPIE in two recent studies. We have reported the approach taken in two novel audiological studies following the GRIPP2 criteria [10]. These criteria guide the reporting of patient and public involvement in health studies. We describe the way PPIE contributed to each of the studies. In each case our aim was to capture a heterogenous range of perspectives. We wanted to include people with differing experiences, cultural backgrounds and clinical experiences. We suspected that these participants were unlikely to reach a simple ‘consensus’ but instead to comment from their own world views on our research plans.

Study 1: The Hearing Loss and Patient Reported Experience Study (HeLP) developed the first ‘Patient-Reported Experience Measure’ (PREM) in audiology [11]. This new measure captures patient experience, burdens and efforts that form daily life with hearing loss (with and without hearing aids). The study involved stages of developing a clear conceptual framework of the ‘experience’ of living with hearing loss, with and without clinical care. Following the development of this conceptual framework, categories were included within the prototype PREM. The prototype questionnaire was subject to test-retest and validation procedures and then implemented into clinical services and evaluated in practice, for detail on this process see Smith et al. 2024 [11].

Study 2: An ethnographic study of adults with dementia and hearing loss in care homes was conducted to understand the factors that contribute to and maintain social isolation among older adults living with dementia and hearing loss. The researchers developed a Grounded Theory model using data from an environmental audit, multiple ethnographic observations, and semi-structured interviews with care home residents, staff members, and relatives across two care homes in Birmingham, the UK [12]. These data were analysed using constant comparative methods in line with Grounded Theory but did not involve use of software.

Both studies were reviewed and approved as conducted in accordance with the principles of the Declaration of Helsinki [13]. The Hearing Loss and Patient Reported Experience study was approved by the West of Scotland Research Ethics Service (22/WS/0057) and the Health Research Authority and Health and Care Research Wales Approval (IRAS project ID: 308816). The ethnographic study of adults with dementia was approved by the West Midlands – Coventry and Warwickshire Research Ethics Committee (19/WM/0294), UK. All participants had cognitive capacity to provide written consent and provided written informed consent prior to participation in the study. ND attended Mental Capacity Act (2005) training to understand the varying presentations of cognitive capacity amongst older adults living with dementia. If a person had fluctuating capacity, informed consent was re-taken at the beginning of every interaction. All research procedures were conducted in keeping with Good Clinical Practice [14].

Specific PPIE activities aimed:

- To prioritise the research questions, PPIE was sought to comment on the validity of research questions to study.
- To seek advice on successful recruitment strategies, respondents contributed to the planning of methods of sampling and recruitment of participants, including planning how to advertise and the type and use of materials to advertise voluntary participation in studies.
- To explore the acceptability of both qualitative and quantitative research methods and activities e.g. interview strategy.
- To ascertain variation and meaningful contrasts in experience (especially among participants with hearing loss who do not seek help in audiology).

## PPIE methods

In order to capture the variation in experience and contrast in public views of our research questions and methods we targeted variation in recruitment of PPIE advice. We recruited individuals who expressed an interest in our research projects from existing groups (volunteer hearing support groups, lip-reading classes, South Asian women’s exercise groups, South Asian religious and community groups, and residential care homes). In addition, we recruited audiology and clinical science students who lived with hearing loss and used word-of-mouth strategies to facilitate introductions to other individuals with views on our work. Participants were invited to engage in key decisions about the research process and were offered £20 vouchers in recognition of their time contributing to our work.

When considering care home populations, PPIE participants were recruited from four care homes in Birmingham, UK. The PPIE respondents were made of care home residents and care home staff. The planning and engagement work was based on building relationships with managers, care staff, and residents. This relationship building is a key part of developing successful working alliances in research [15].

**Fig. 1:**
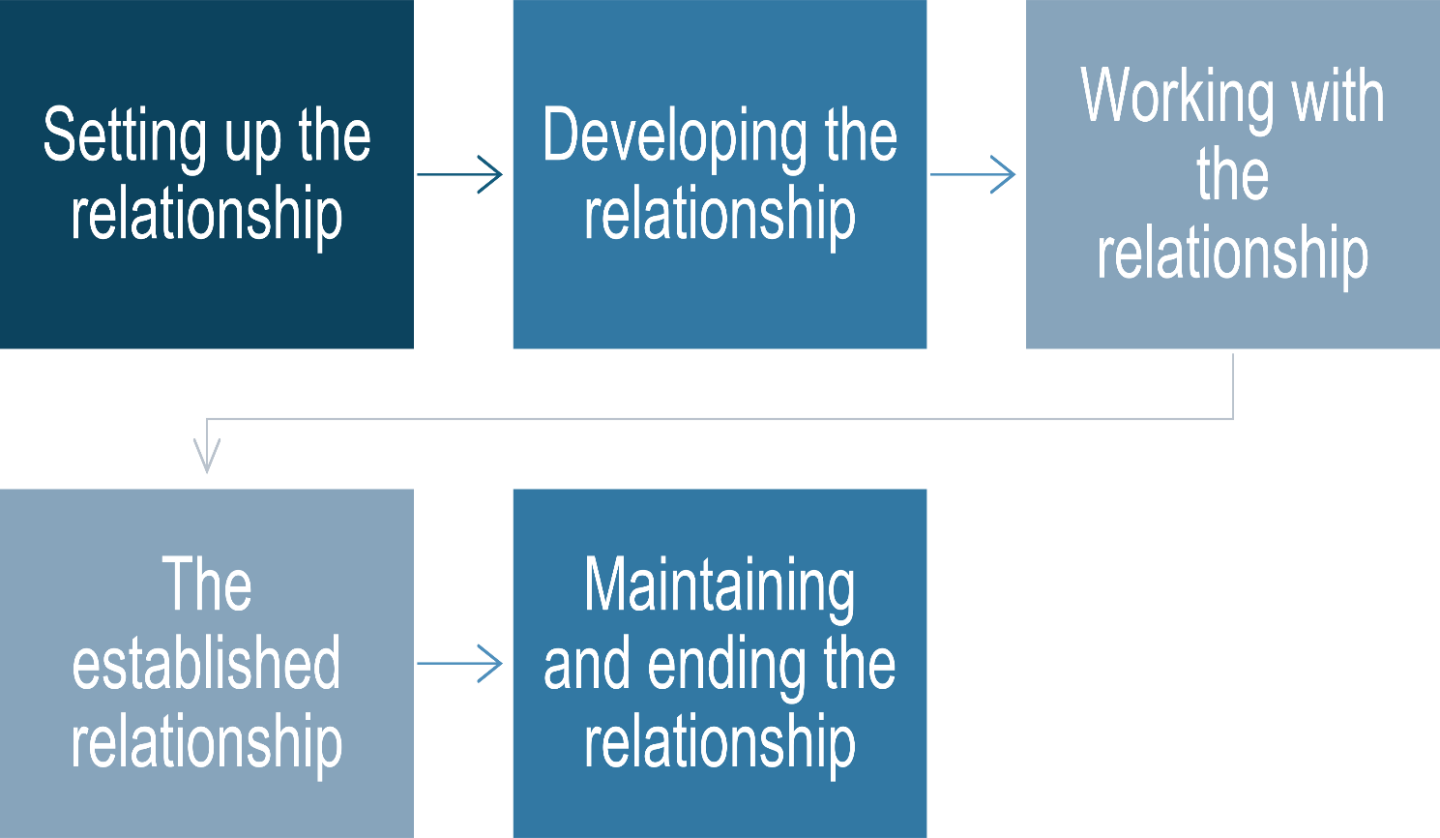
Relational skills model steps by Midwinter and Dickson (2015) [16]

The researcher (ND) met with everyone who expressed an interest in the proposed research and held individual meetings in residents’ rooms and, for staff, in office areas of the care homes. These PPIE encounters were informed by the relational skills model (see Fig. 1) developed by Midwinter and Dickson (2015) [16] to facilitate effective communication. The model details the five phases involved in developing a helper relationship. This follows a continuum of setting up the relationship to ending and maintaining the relationship. The philosophical underpinnings of this model are derived from the Rogerian approach to person-centred therapy [17]. In this approach, the core conditions needed for the free exchange and expression of information, especially aimed at change, require congruence, empathy, and unconditional positive regard. Expressing these qualities can lead to a person feeling empowered to meaningfully contribute to a situation based on their life experiences [17]. Empowerment is at the core of this model and therefore fits for use with marginalised communities [18]. The lead facilitator was trained in the model as part of a postgraduate Hearing Therapy programme that taught communication and therapeutic skills.

### Including marginalised groups

South Asian community groups (affiliated with religious and spiritual centres) were notified about the research via word of mouth, initiated by one researcher of Punjabi descent. These groups were based in Birmingham, UK, and were community hubs within the South Asian communities of Birmingham. The leaders of the groups cascaded information about the purpose and importance of the PPIE and the voluntary nature of the activity. Individuals interested in participating contacted the researcher via email, in response to word-of-mouth interactions amongst community members.

Student and staff at Aston University with lived experience of hearing loss were invited via advertisement to contribute to feedback on the HeLP study. Volunteering individuals were contacted via email and contributed feedback at each stage of the research process via email. Volunteers at clinical services in Bath (mostly older adults) were contacted by the Chief Investigator (HP) at their existing volunteer meetings and were invited to contribute views. Their contributions then took place individually and they contributed via online meetings and email.

In addition, individuals responded to advertisement (including via word of mouth) and via X (formerly Twitter), Facebook, online advertisement etc. Finally, members of lipreading classes at different and contrasting postcode districts of Bristol were invited to contribute PPIE feedback. The chief investigator visited classes and during breaks in the class, the class members provided contributions as a focus group. In total approximately 40 individuals provided PPIE steering in this way.

PPIE respondents were recruited from within the care home communities of Birmingham where the research was set. Care homes that specialised in dementia care were contacted either by email or telephone, to the attention of the care home manager. In total 20 care homes were contacted, of which four were willing to participate. The homes that did not want to be involved cited reasons such as lack of capacity to conduct research, no interest in research participation, or no residents meeting the inclusion criteria of the study. The four homes that did respond provided a broad range of socio-economic and demographic status within Birmingham, determined by Acorn profiling of residential postcode [19, 20]. Six people contributed PPIE steering.

**Table 1:** Aims of PPIE activities for studies 1 and 2, and questions asked to PPIE informants for each aim

Aim of PPI activities	Questions Asked
Study 1: To understand the experiences of people from marginalised communities about their interaction with Audiology and hearing services	• Tell me about your experience of hearing loss. • What is your experience of Audiology services? • What do you think works and what does not? • Did you have any experience of people with a hearing loss as a child? • How has your perception of hearing and hearing loss changed over time? • Have you thought about this in relation to yourself? • Any surprises about living with hearing loss? • What do researchers interviewing people with hearing loss need to know? • How should researchers approach interviews? • The purpose of the research will be do develop a questionnaire about your experiences. How would you feel if we gave you another questionnaire to fill in? • When would you prefer to fill it in? • What is the best way to measure your experience?
Study 1: To establish whether the developing PREM is culturally meaningful, acceptable, and accessible to a South Asian population	• Do you relate to the questions? • How would these questions be perceived in your culture? • Are you able to discuss your hearing experiences openly in your culture?
Study 1: To establish the translatability of the proposed PREM to South Asian languages	• Are there any words or phrases that are not directly translatable? • Can you think of alternative phrases that may be more appropriate?
Study 2: To establish willingness to participate	• How would you feel about being observed? • What is the level of involvement of relatives in the care of residents?
Study 2: To establish knowledge on the relevance and importance of the proposed ethnography	• What have you noticed about the interactions of residents with hearing loss and dementia? • What are your thoughts on improving social isolation within the home? • What were the main barriers to improving social isolation? • Have you noticed any hearing difficulties in residents living with dementia?
Study 2: To establish an understanding of staff attitudes towards the proposed projects	• What type of training have you received to effectively communicate with residents who have hearing impairment and/or cognitive impairment/dementia? • What would you like introduced within the home to help you to communicate more effectively with residents?

### Gathering perspectives

PPIE groups (e.g. the volunteer group in Bath and student/staff members at Aston) were asked about what aspects of living with hearing loss they thought were and were not well understood by their clinicians in audiology. We asked open questions such as ‘what is important to you about living with hearing loss?’ ‘What do you think people should know about living with hearing loss?’ ‘What do you think clinicians should know about hearing loss?’. This led to responses which highlighted the residual difficulties people have after hearing aids are fitted. The discussions led to the importance of clinicians knowing what it is like for people with hearing loss. PPIE respondents emphasised the amount of hidden labour that having a hearing loss entails, the repeated efforts to manage communication in noise for example, is an on-going effort which hearing aids often do little to improve.

An environmental audit of each care home was conducted by ND (an audiologist by training) to identify the acoustic properties and possible hearing barriers of communal spaces. ND gathered an understanding of a resident’s ability to listen at normal conversational level with background noise through their responses and facial expressions when conversing in communal areas of the home. To enhance the conversational setting, we proposed quieter venues, such as the dining room (excluding mealtimes) or the library, where potential PPIE participants expressed interest in more in-depth discussions.

Over the next 3 months, we made weekly visits to the homes and the residents began to recognise us as researchers. The relationship was developing, and we started to become familiar within the settings. This meant that we could start to ask specific questions about the current research ideas to individual residents who chose to contribute to the planning and engagement.

At the initial meeting with each care home manager, we sought to determine an overview of the type of residents, staff, and general culture of the home. In accordance with the aims and objectives of the PPIE work, we asked questions that aimed to identify the amount of variation within and between care homes, to identify important contextual differences that would influence potential research participation. For example, to identify numbers of residents with dementia who had capacity to participate in research etc. This enabled the researcher (ND) to gather an understanding of the care home demographics. The level of detail and enthusiasm to the answers provided gave ND an indication of willingness and acceptance of research within the environment.

Following the initial meetings, monthly visits were arranged where we spent time observing the routine functioning of the care home to better understand the role of care staff. This was the necessary preparatory work for the proposed ethnography and interviews, as an understanding of carer roles and duties would have a direct impact on the time they had available (and willingness) to take part in research. Care staff were consulted about the proposed research to find out their views on its relevance and importance. Furthermore, residents were consulted individually about their experiences of living in care homes and what their daily activities usually consisted of, to understand their current level of engagement and satisfaction. The questions in Table 1 were used to guide the discussions. The researcher spent approximately four hours at each home per month.

Initial communications with care home managers were formal via email or telephone and were used to “set-up” the relationship. This process involved contacting and meeting the person using attending, active listening, and contracting skills. Care home managers were viewed as the “gatekeepers” to conducting the proposed research. We used the first meeting with each care home manager to summarise my previous employment and qualifications, current job role, and rationale for the ethnographic research. Thus, we started to develop the relationship (second stage of the relational skills model) using presence and effective communication (see Fig. 1).

There is often apprehension about external visitors watching the day-to-day running of residential care settings [20], which usually equated to inspections such as from the Care Quality Commission (the independent regulator of health and social care in England). The relationship between the researchers and care staff became more substantial and more developed when the researchers were transparent about their agenda and willing to adapt the research methods to suit the needs and requirements of the home. It was essential to emphasise the intention to not judge or criticise but instead observe and listen. This was an essential component in helping to form relationships and develop a working alliance based on mutual respect and understanding.

We spoke with staff members individually about the proposed research to discern their views on its relevance and importance, and to understand what was currently in place to help residents engage. We also spoke with the residents about their experiences of living in care homes and their daily activities, aiming to understand their current level of social interaction and general satisfaction. This stage was ‘working with the relationship’ and practising healthy boundaries and behaviours in terms of how much of their time we took and the level of detailed questioning [16].

After the initial meetings, the researcher visited the homes more frequently (approximately once a month). These visits served as invaluable opportunities to gain deeper insights into the daily operations of the care home and to observe the routines of both the residents and staff. The primary objective during these visits was to identify any instances of social isolation that were prominent within the care home environment. By embedding into day-to-day activities such as mealtimes, spending time in communal areas to watch television and read books, and structured group activities, the researchers aimed to uncover the nuanced aspects of resident interactions and detect subtle signs of isolation that might not be immediately apparent. For example, non-verbal communication cues, such as body language, facial expressions, or gestures, often feelings of loneliness or disconnection. In addition, there was a lack of engagement and conversation with other residents and staff members, and no observed visits from family or friends. There were also expressions of sadness and frustration during limited interactions with staff or fellow residents, suggesting underlying emotional distress or loneliness [12].

Furthermore, these visits provided an excellent vantage point to better understand the pivotal role of caregivers. Through direct observation, the researchers sought to comprehend the dynamics of caregiver-resident interactions, the quality of emotional support provided, and the challenges faced by caregiving staff in mitigating social isolation among residents. This hands-on approach not only enriched the understanding of the social dynamics within the care home but also established an understanding with the caregivers, fostering a collaborative environment for sharing insights and experiences.

### Results of PPIE – lessons for audiology research

**Table 2:** The purpose and function of PPIE – our plan to optimise engagment

Aim of Public and Patient Involvement and Engagement	Action	Result: • Changes to research methods and approach
• Engage a heterogeneous range of people affected by hearing loss	• Target specific marginalised communities through engagement in community settings • Flexible interview and participation arrangements • Build a relationship	• Empathy and acceptance of different views • Flexibility in recruitment strategies
• Seek advice on prioritising research questions • Research method application	• Which questions are most meaningful? • How should we sample and recruit participants? • Have we interpreted findings in a way that has face validity?	• Be mindful of burdens of participation and minimise these • Assist interpretation of data • Refine writing

### Methods guidance

Our PPIE informants guided our approach to gathering interview data. Specifically, we were advised to demonstrate the following qualities.

- **Empathy** – There was a perceived need for researchers to approach participants in a non-judgemental way and respect their views of their hearing. This included not judging people who chose not to seek help or to use devices to help their hearing.
- **Acceptance** is considered important to communicate during interview including respecting language used (e.g. not referring to hearing loss and hearing aids but respecting variation in language participants use to describe their situation). Our PPIE respondents proposed that researchers listen to the terms used to describe individual conceptions of their hearing and reflect the chosen language used e.g. while some people refer to themselves having ‘hearing loss’, others refer to their ‘hearing’ without conceptualising it as a loss, rather a difference.
- **Cultural sensitivities** – discussion about stigma of hearing loss and importance of not imposing medicalised language in interviews. In some cultural groups it is particularly important not to emphasise a medicalised ‘loss’ of hearing and the stigma that follows from being described as ‘impaired’ would be considered a barrier to participation in research. We adapted and re worded materials to avoid referring to ‘loss’ in participant materials.
- **The burdens of participation**. Our informants referred to concerns about the amount of time that participation in the study would take. We were advised to be mindful of only involving people as necessary and not taking more time out for travel to interviews etc. As a result, we shifted our approach to travel to respondents and interview participants and made all engagement possible via online platforms. We also organised our PPIE involvement so that specific points were taken to each group and coordinated by the PPIE lead (author J.S.) and researchers.

Care staff participated in interviews with the researcher around their tasks in looking after the residents. Interviews were scheduled during less busy periods, but flexibility was maintained for the participant’s convenience. Caregivers reported that residents are often more likely to participate in activities if their acquaintances are also present, but conversations may still be absent due to cognitive demands or inappropriate environments. Therefore, activities were a significant focus in the ethnographic observations to better understand resident interactions. One-on-one interactions between staff and residents were useful for observations, but these were influenced by the acoustic environment of the home. Care homes with low ceilings, carpeted floors, and soft furnishings are more conducive to communication for individuals with hearing loss, while homes with high ceilings, hard floors, or wooden furniture may be more challenging. During our visits, we noticed the conflicting sound sources in communal areas, which may discourage residents with hearing loss from engaging in conversation and socialising. These findings emphasised the importance of providing an appropriate hearing environment for residents when they are in communal areas, which was discussed with care home staff.

### Interpretation of data

To comment on our emphasis and selection of themes in the data our PPIE lead (author JS) participated in analysis sessions where researcher presented direct quotations and described the researcher interpretations of these comments. She was able to see the process of coding meaning statements in data and then grouping codes. These findings were fed back to the older age PPI group in Bath and younger PPI group in Birmingham. She was able to communicate our decisions and was receptive to commentary on language and interpretation of findings - she fed this back to the research group.

We worked closely with our PPIE lead in considering language used to interpret and describe qualitative themes in our data sets. JS highlighted ambiguities in the labelling of qualitative data categories derived from the research process.

To comment on the clarity and transparency of our decision making throughout. PPIE contributors brought us comments on the way to approach, engage, and interview participants. They have provided helpful insights into reaching marginalised groups, recruiting younger people and engaging with clinicians.

Our PPIE lead (JS) attended and contributed to our steering group and provided a scrutiny of our process.

### PPIE commentary on data findings

PPIE respondents raised concerns about any research activities that would lead to increases in clinical ‘paperwork’ and challenges of increasing (or being seen to increase) ‘paperwork’ for service users (especially older adults in residential care settings). This informed our decision to aim for reducing additional paperwork in administration of our PREM and to reduce the scale of the PREM to as few items as possible. Our themes on the individualised nature of hearing loss resonated for participants. Common comments were how important it is to speak to people directly affected by hearing loss, how challenging it is to live with hearing loss and feel lonely or mis-understood - even by close family. Our research findings were described as making sense and chiming with the lived experience of PPI members. In particular the extent to which the individual with hearing loss feels alone in managing it without support in day-to-day decision making or adjustment. Members of local groups and lip-reading classes noted that the support they got from groups they had sought out was invaluable in overcoming this lonely experience.

There were some staff concerns expressed that extra work would be added by participating in research. This informed the way that research was conducted in the care homes settings.

The views of PPIE informants were largely considered and helped to shape the methods and design of the ethnographic work. In addition to question formulation, the PPIE informants helped to steer the time and conduct of interviews and observations, and the optimal recruitment approach for resident and relative participants. Importantly, potential participants spoke positively of the work and felt the findings would be highly beneficial to them. Similarly, most staff members said they would be happy to be observed if the observations did not disrupt their usual work routine or delay their duties. Being discreet and self-contained during the observations reduced the potential of reactivity from those in the research setting [21] and put care staff at ease. Furthermore, interviews were arranged at the beginning or end of a shift, or during a period of low activity in the home, for minimal disruption to care staff duties, especially as time was mentioned as a barrier to participation. When asked, the staff members who appeared reluctant to participate were assured that their participation in the research was wholly voluntary and that they could leave the study at any time without being questioned. This was written clearly on the participant information sheet and reiterated verbally to them multiple times.

### PPIE contribution and iterative refinement to PREM items

Over a span of 26 weeks, 56 individuals with a vested interest in hearing loss generously provided feedback to fine-tune the prototype PREM questions. Through a blend of in-person meetings, online platforms, phone calls, and virtual gatherings, we invited our PPIE members to shape the evolving PREM drafts. Our questions to them were designed to check the face validity of the items prior to psychometric testing.

- Do these questions mirror your lived experience of hearing loss?
- How do these questions deviate from the clinical norm?
- What crucial elements are we overlooking?
- What should take precedence in our inquiry?
- Is the language accessible and comprehensible?
- Is the overarching purpose of our investigation evident?
- Would you be inclined to complete this questionnaire?

The PPIE feedback advised us that:

- The resonance of the PREM items with the experiences of PPIE members affirmed their relevance.
- PPIE participants appreciated the departure from conventional clinical questioning, acknowledging the focus on residual challenges.
- Their response to our invitation to prioritise highlighted gaps in earlier iterations, prompting the inclusion of new items addressing public-facing hurdles and communication obstacles with service providers.
- Their feedback on the wording of items paved the way for clarity enhancements, steering clear of language that might reinforce negative perceptions.
- Through an iterative process, we meticulously reworked 39 items, eliminating ambiguities and ensuring inclusivity and clarity in language.

Finally, it’s worth noting that every one of our PPIE collaborators actively contributed to refining the PREM, completing both the questionnaire and the subsequent validation survey, which encompassed a broad array of measures capturing communication function, loneliness, health literacy, decision-making, quality of life, and capacity [11].

## Discussion

This paper describes a novel approach to PPIE contribution to audiological research. Rather than aiming for consensus opinion gathering, we have prioritised relationship building to ensure a wide range of views and voices within our research programmes. Our approach celebrated variation and included often marginalised community members from communities with a disproportionately high prevalence of hearing loss (care home residents; South Asian community members etc.). Following guidance of psychotherapeutic models, we have prioritised relationship and concurrent free expression of opinion as the active ingredient to find out how best to conduct research [16]. Ocloo et al. (2021) [22] emphasise the necessity of a nuanced understanding of diversity in the context of patient and public involvement. Our approach draws inspiration from this perspective, striving to amplify the voices of those who often face barriers to participation. Their systematic review highlights the inadequacies of traditional focus groups in capturing the intricate tapestry of experiences within marginalised communities. By adopting a more personalised and relational approach, we aim to bridge this gap, fostering a deeper connection that transcends the limitations of traditional focus group methods.

Oftentimes, the exclusion of marginalised communities in traditional focus groups can be attributed to the way PPIE initiatives are advertised and targeted [23]. This underlines the need for innovative strategies that actively reach out to underserved populations. The significance of community participation and the concept of “snowballing” in engaging marginalised communities with limited access to healthcare has been recognised as a pivotal success factor in promoting representative PPIE [24]. By deliberately seeking out and engaging with these often-overlooked communities, we aimed to address the limitations inherent in traditional focus groups and fostered a more comprehensive understanding of diverse perspectives in our research.

Implementing personalised and relational approaches in PPIE presents several challenges and limitations that researchers must navigate to ensure the effectiveness and integrity of their methods. One primary challenge is the resource constraints associated with conducting intensive community engagement activities and building trust-based relationships. This often requires dedicated funding, time, and personnel to establish and maintain meaningful connections with diverse communities [25]. The time-intensive nature of personalised and relational approaches can pose logistical challenges, particularly in research projects with tight timelines or limited resources [26]. Building trust and rapport with marginalised communities often requires ongoing commitment and sustained engagement over an extended period, which may not always align with the constraints of traditional research timelines [27].

Additionally, researchers must be mindful of potential biases introduced by their own perspectives, backgrounds, and positionalities when engaging with communities. This includes being aware of power dynamics and ensuring that research activities are conducted in a culturally sensitive and respectful manner [28]. Therefore, researchers should prioritise transparency and reflexivity throughout the research process to mitigate potential biases and power imbalances. This involves acknowledging and critically examining their own positions of privilege and seeking input and feedback from community members to ensure that research activities are conducted ethically and inclusively [29].

## Conclusions

Our approach prioritised relationship-building instead of achieving consensus, recognising the value of diverse perspectives in enhancing research outcomes. By incorporating the views of often marginalised community members, we addressed the limitations of traditional PPIE methods and promoted a more inclusive and representative practice. Overall, our study contributes to the growing body of literature advocating for more inclusive PPIE practices in health research. It suggests that flexible and personalised engagement methods can lead to more relevant and impactful research. The research projects were informed by several key pieces of PPIE. Future research should continue to explore innovative strategies for involving a wide range of public contributors, ensuring that all voices are heard and considered in the research process. This approach will ultimately enhance the quality and relevance of audiology and other health-related research, leading to better outcomes for all stakeholders.

## Data Availability

No datasets were generated or analysed during the current study.

## Abbreviations

HeLP: Hearing Loss and Patient Reported Experience Study
PPIE: Patient and Public Involvement and Engagement
